# A robust circadian rhythm of metabolites in *Arabidopsis thaliana* mutants with enhanced growth characteristics

**DOI:** 10.1371/journal.pone.0218219

**Published:** 2019-06-25

**Authors:** Dieuwertje Augustijn, Huub J. M. de Groot, A. Alia

**Affiliations:** 1 Leiden Institute of Chemistry, Leiden University, RA Leiden, The Netherlands; 2 Institute of Medical Physics and Biophysics, University of Leipzig, Leipzig, Germany; Karlsruhe Institute of Technology, GERMANY

## Abstract

Climate change and the rising food demand provide a need for smart crops that yield more biomass. Recently, two *Arabidopsis thaliana* mutants with enhanced growth characteristics, VP16-02-003 and the VP16-05-014, were obtained by genome-wide reprogramming of gene expression, which led to the identification of novel biomarkers of these enhanced growth phenotypes. Since the circadian cycle strongly influences metabolic and physiological processes and exerts control over the photosynthetic machinery responsible for enhanced growth, in this study, we investigate the influences of the circadian clock on the metabolic rhythm of eighteen key biomarkers for the larger rosette surface area phenotype. The metabolic profile was studied in intact leaves at seven different time points throughout the circadian cycle using high-resolution magic angle spinning (HR-MAS) NMR. The results show that the circadian rhythm of biomarker metabolites are remarkably robust across wild-type Col-0 and VP16-02-003 and the VP16-05-014 mutants, with widely different metabolite levels of both mutants compared to Col-0 throughout the circadian cycle. Our analysis reveals that robustness is achieved through functional independence between the circadian clock and primary metabolic processes.

## Introduction

Smart crops with high biomass yield and with a reduced need for fertilizer and pesticides can help to meet the increasing demand for agricultural products [1,2]. Such smart crops can be developed by genome editing tools including zinc finger nucleases (ZFNs), transcription activator-like effector nucleases (TALENs) and clustered regularly interspaced short palindromic repeats/Cas9 (CRISPR/Cas9) [3,4]. In a recent study, zinc finger artificial transcription factors (ZF-ATFs) were used to obtain *Arabidopsis thaliana* mutants with enhanced growth characteristics [5]. The transcriptional activator protein VP16 from the herpes simplex virus was fused to an array of three zinc fingers (3F) to provide an artificial gene construct, denoted 3F-VP16. *Arabidopsis thaliana* plants were transformed with the 3F-VP16 construct under the control of the RPS5A promotor. The 3F motif has ~1000 binding sites in the nuclear *Arabidopsis thaliana* genome of 130 Mbp. This could lead to drastic changes in genome-wide expression patterns and provide access to rare phenotypes of plants [4,6]. Indeed two *Arabidopsis thaliana* mutants, VP16-02-003 and VP16-05-014, with enhanced growth characteristics were obtained using such genome interrogation with ZF-ATFs [5].

Recently, we have also introduced a systems biology approach to resolve primary processes of metabolic regulation and conversion leading to enhanced growth from the complicated biological background, using non-invasive HR-MAS NMR [7]. In the previous work, the metabolic profile for both mutants was studied at one time-point in the middle of the light period of 12 hours in a 24-hour light/dark regime [8]. The changes in the metabolomics and transcriptomics of both mutants compared to the wild-type *Arabidopsis thaliana* accession Columbia-0 (Col-0) gave us insight into the improved growth characteristics of the mutants which were found to be a consequence of the reduce defence response in the context of the growth-defence trade-off [5,8–11].

**Fig 1:**
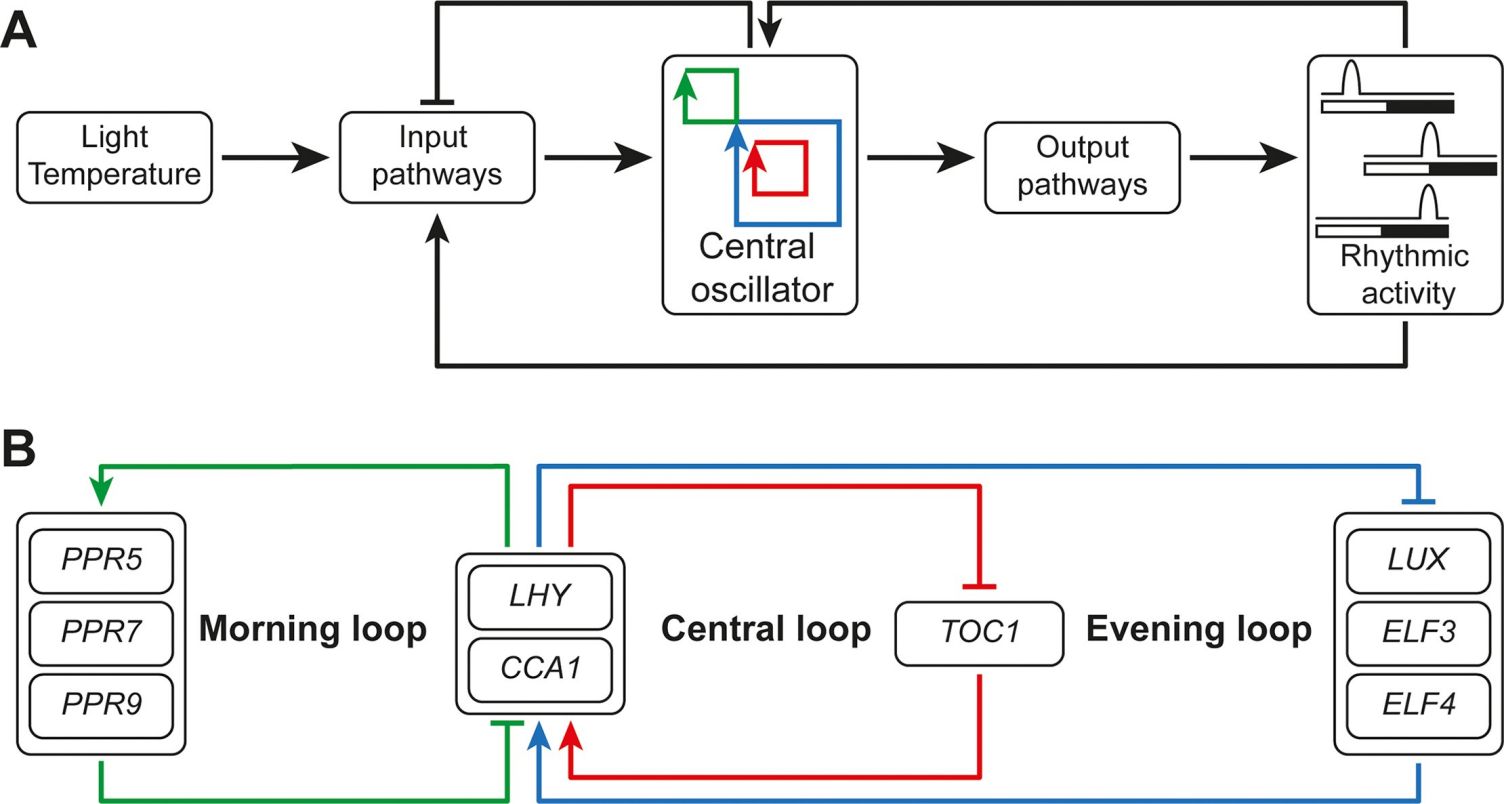
Schematic overview of the *Arabidopsis thaliana* circadian clock. A) The central oscillator consists of three interlocked feedback loops. External signals like light and temperature can influence the input pathways for the central oscillator. In turn, the central oscillator activates output pathways depending on the time of the day. B) Model of the central oscillator containing three interlocked transcriptional-translation feedback loops.

It is known that physiological functions, like growth, flowering time, response to stress and metabolism are regulated by the circadian cycle [9,10]. A circadian cycle is a biological process that displays an endogenous, entrainable oscillation of about 24 hours. Hence the circadian clock comprises three physical entities that fulfil the functional requirements of pre-dawn activation, evening deactivation and the afternoon switching between the morning and evening regimes, providing a nice example of a function based framework with limited complexity (Fig 1A) [11]. The molecular mechanism of the circadian clock in *Arabidopsis thaliana* has been studied extensively during the last decades and consists of three interlocked transcriptional-translation feedback loops (Fig 1B) [9,10,12–14]. The central loop of the circadian clock consists of the morning-expressed genes *CCA1* (*CIRCADIAN CLOCK ASSOCIATED 1*) and *LHY* (*LATE ELONGATED HYPOCOTYL*) and the evening-expressed gene *TOC1* (*TIME OF CAB EXPRESSION 1*). CCA1 and LHY form a heterodimer which represses the expression of *TOC1*. The heterodimer also positively regulates the afternoon-expressed pseudo-response regulators PRR5, PRR7 and PRR9. In turn, PRR5, PRR7 and PRR9 repress the transcription of CCA1 and LHY, which allows the induction of the evening-expressed gene *TOC1*. The evening-expressed genes are *ELF3* (*EARLY FLOWERING 3*), *ELF4* and *LUX* (*LUX ARRYTHMO*) and their products form the evening complex (EC). The evening complex represses the expression of the PRRs which allows the expression of CCA1 and LHY in the early morning [9,10,12–14].

According to recent insights into the biological design from a functional perspective, robustness requires functional independence of the abundant subsystems, which, in the case of the clock, are controlled through interfaces while leaving the clock itself undisturbed [11]. In this work, we show that this is in fact realized in the *Arabidopsis thaliana* VP16-02-003 and VP16-05-014 mutants, where gross changes in concentrations of primary metabolites are realized while the clock is running in phase across wild-type and mutants.

We have studied the circadian rhythm of the metabolites using high-resolution magic angle spinning (HR-MAS) NMR to obtain the metabolic profiles at seven different time-points of the light/dark cycle for the *Arabidopsis thaliana* wild-type Columbia 0 (Col-0) and two mutants, VP16-02-003 and VP16-05-014. The data provide converging evidence that the clock functional periodicity is independent of mitigation of cellular complexity and growth-defence trade-off.

## Materials & methods

### Plant materials and sample collection

*Arabidopsis thaliana* accession Columbia-0 and VP16-02-003 and VP16-05-014 plants were grown as described previously [8], with slight modifications. Plants were grown in a climate-controlled growth cabinet (Bronson climate BV) instead of green house used in earlier study [8]. Use of growth cabinet was necessary to achieve controlled harvesting of the leaves at various time points during light/dark cycle. Plants were grown at 293 K, 70% relative humidity and with a light regime of 12 hours light (200 μmol m^-2^ s^-1^ provided by fluorescent lamp) and 12 hours dark. In the growth chamber, the plant growth was slower than in green house, therefore rosette leaves were harvested at 35 instead of 28 days post germination to achieve the same growth stage. Typically, samples of intact rosette leaves were harvested every 4 hours at seven different time-points throughout the light/dark cycle. For HR MAS NMR, each fresh single rosette leaf was weighed (n = 6) and rolled inserted into a 4 mm ZrO_2_ rotor with 10 μL of deuterated phosphate buffer (100 mM, pH 6) containing 0.1% (w/v) 3-trimethylsilyl-2,2,3,3-tetradeuteropropionic acid (TSP). The sample rotors were immediately frozen in liquid nitrogen and stored at -80˚C until use. Samples for quantification of free amino acid, protein, sugars and starch were weighed and frozen immediately in liquid nitrogen and stored at -80˚C until use. The VP16-02-003 and VP16-05-014 mutant with an increased rosette surface area phenotype were investigated in this study in comparison to the wild-type *Arabidopsis thaliana* accession Columbia-0 (Col-0) [5].

### Quantification of free amino acids, proteins, soluble sugars and starch

For the estimation of free amino acid content of the leaves, the amino acids were extracted from leaves (~60 mg) using 600 μL 80% (v/v) ethanol at 80°C for 30 minutes. The sample was centrifuged for 15 minutes at 18.000 g at 4°C. The supernatant was collected and the pellet was suspended in 400 μL of 50% ethanol and incubated for 20 minutes at 80°C. The suspension was then centrifuge for 15 minutes at 18.000 g at 4°C. The supernatant was again collected and pellets were resuspend in 300 μL water. Prior to centrifugation, the suspension was again incubated for 20 minutes at 80°C. After centrifugation, all the supernatants were pooled and used for further analysis. The free amino acid content was assayed calorimetrically using 8% (w/v) ninhydrin solution as described earlier [15]. For protein estimation, proteins were extracted from leaves (~30 mg) by using 60 μL ice-cold extraction buffer containing 100 mM Tris-Cl (pH 7.2), 2 mM MgCl_2_, 2 mM EDTA, 5 mM DTT, 2% (m/v) PVPP and 10% (v/v) glycerol). The sample was stirred for 15 minutes at 4 ˚C and the extract was centrifuged for 5 minutes at 10.000 g at 4 ˚C. The supernatant was used to determine the protein content using a Bradford assay [16]. For estimation of soluble sugars, the same ethanol extraction method as for free amino acids was used. The amount of total soluble sugars was estimated according to the procedure of Dubois et al. [17]. Briefly, suitably diluted alcohol extracts were mixed with 5% (w/v) aqueous phenol and concentrated sulphuric acid. The contents were incubated for 30 min at room temperature and absorbance was read at 490 nm. The starch contents were determined by following the methods as described by Smith and Zeeman [18]. Briefly, the starch in the ethanol extracts were first converted into glucose using α-amyloglucosidase and α-amylase. Subsequently, the amount of glucose was determined by using hexokinase and glucose-6-phosphate dehydrogenase [18].

### Fumarase activity assay

To assay the fumarase activity, a colorimetric assay kit of Sigma-Aldrich (MAK206) was used. Enzyme extract were prepared by homogenizing leaves (~10 mg) in 100 μl ice cold Fumarase assay buffer (Sigma-Aldrich MAK206A). After 10 minutes of incubation on ice, the extract was centrifuged at 10,000 x g for 5 min. Supernatant was used for the fumarase activity assay. Fumarase activity was determined by measuring a colorimetric product with absorbance at 450 nm (A450) proportional to the enzymatic activity present by using Emax Plus microplate reader (Molecular Devices) in the kinetic mode.

### Statistical analysis

The one-way analysis of variation (ANOVA) function of OriginPro 2016 software (Northampton, USA) was used to determine the significant differences between *Arabidopsis* wild-type Col-0, VP16-02-003 and VP16-05-014 for the data for each experiment. Values are presented as means ± standard error (SEM) and statistical significances were determined at p < 0.05 for Col-0 vs VP16-02-003 and for Col-0 vs VP16-05-014 adjusted for multiple testing using the Holm-Bonferronie correction method.

### HR-MAS NMR-based metabolic profiling

A Bruker AV-400 MHz spectrometer operating at a resonance frequency of 399.427 MHz was used for the HR-MAS NMR experiments with a 4 mm HR-MAS dual inverse ^1^H/^13^C probe with magic angle gradient. A spinning frequency of 4 kHz and a temperature of 277 K was used during data acquisition.

A rotor synchronized Carr-Purcell-Meiboom-Gill (CPMG) pulse sequence with water suppression was used to obtain one-dimensional ^1^H HR-MAS spectra [19]. The one-dimensional spectra were collected applying 256 transients, a spectral width of 8000 Hz, data array size of 16K points, an acquisition time of 2 seconds and a relaxation delay of 2 seconds. The echo time applied for CPMG-spectra was 6.4 ms (8 loops, rotor-synchronized inter-pulse delay of 0.8 ms). The free induction decays (FIDs) were exponentially weighted with a line broadening of 1 Hz. TOPSPIN 3.5 (Bruker BioSpin, Germany) was used to phase the spectra manually and to perform automatically baseline correction. Data deposit publicly available.

### Multivariate analysis

AMIX (version 3.8.7, Bruker BioSpin) is used to generate bucket tables for each time-point from the one-dimensional spectra excluding the region between 4.20–6.00 ppm to remove the larger water signal. The one-dimensional CPMG spectra were normalized to the total intensity and binned into buckets of 0.04 ppm. The data were mean-centred and scaled using the Pareto method in SIMCA software package (version 14.0, Umetrics, Umeå, Sweden). Supervised orthogonal partial least squares discriminant analysis (OPLS-DA) was performed on the data using the SIMCA software.

### Quantification of the metabolites

The eighteen metabolites were quantified using the Chenomx NMR Suite 8.2 (Chenomx Inc., Edmonton, Alberta, Canada). The known concentration of the reference peak of TSP was used to determine the concentration of the eighteen biomarkers. Metabolite concentrations are represented as means ± standard error. Student's t-test analysis of the NMR quantification results was performed with OriginPro 2016 (Northampton, USA).

## Results and discussion

### Circadian rhythm of amino acids, proteins, sugars and starch

**Fig 2:**
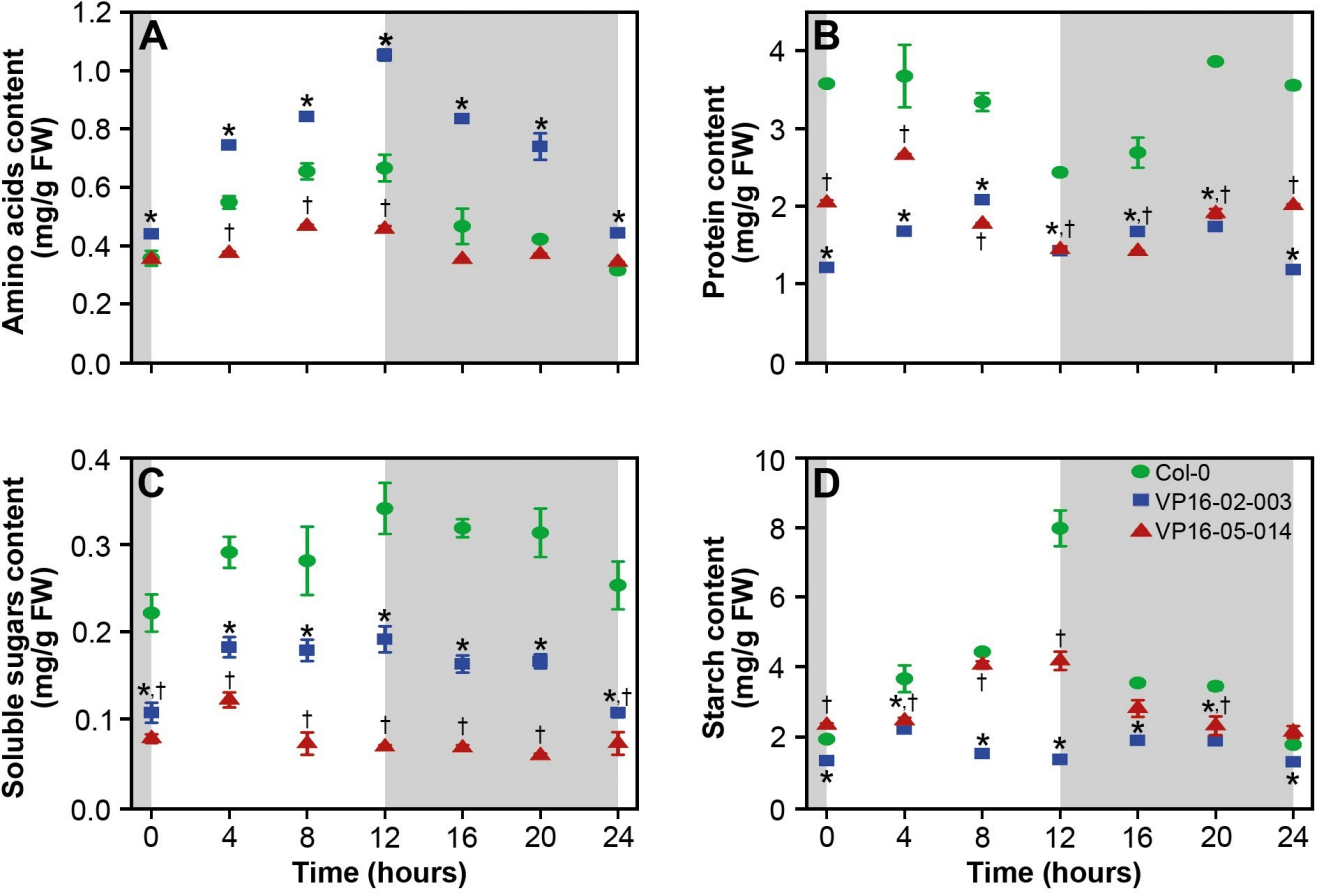
Changes in concentration of free amino acids (A), proteins (B), soluble sugars (C) and starch (D) throughout the circadian cycle for *Arabidopsis thaliana* Col-0 (●), VP16-02-003 (◼) and VP16-05-014 (▲) in mg/g fresh weight. The results are given as mean ± SEM (n = 6). * p < 0.05 Col-0 vs VP16-02-003, ^†^ p < 0.05 Col-0 vs VP16-05-014.

The functional pathways underlying the enhanced growth characteristics of the VP16-02-003 mutant and the VP16-05-014 mutant were investigated in this study throughout the circadian cycle. Prior to metabolic profiling, the circadian rhythms for the free amino acids, proteins, soluble sugars and starch were examined (Fig 2).

In the wild-type Col-0, the free amino acid concentration shows an increasing trend during the light period, while levels drop during the dark period, in line with earlier studies (Fig 2A) [20]. The variation of the concentrations in both mutants followed the same rhythmic pattern as for the Col-0. The metabolite concentrations of the amino acids were significantly higher in VP16-02-003 than for the Col-0, on the other hand, the concentration of free amino acids for the VP16-05-014 mutant was slightly lower at the beginning of the light period and very similar to the Col-0 in the dark period. The concentration of proteins remained constant throughout the light/dark cycle except for a dip at the beginning of the dark period in Col-0 as well as in both mutants (Fig 2B). However, the level of proteins was significantly lower for the VP16-02-003 and VP16-05-014 mutant relative to Col-0 throughout the light/dark cycle.

In photosynthesis, CO_2_ is assimilated into sugars that are used for several primary processes in plants. Starch is utilised during the dark period [21,22]. For both sugars and starch, we expect a rhythm where the compound has risen to a maximum at the end of the light period and reduction takes place during the dark period [21,22]. The concentration of sugars in the *Arabidopsis thaliana* Col-0 show a maximum level at 12 hours and reduced during the dark period (Fig 2C). The VP16-02-003 mutant follows the same rhythm however, the concentration of soluble sugars was lower throughout the circadian cycle as compared to Col-0. The concentration of soluble sugars was found to be constant for the VP16-05-014 throughout the light/dark cycle. In Col-0, the starch level was elevated during the light period and dropped during the dark period (Fig 2D). The starch content of the VP16-02-003 mutant remained at a lower level in contrast to Col-0 during the whole period and this mutant does not show a high concentration of starch at the beginning of the dark period. VP16-05-014 follows the same rhythm as Col-0 but with a lower amplitude. These results suggest that most probably, soluble sugars and starch are not the most favourable carbon storage possibility in these mutants. Organic acids, like fumaric acid and malic acid, can be used as an alternative carbon storage in *Arabidopsis* plants [23–25]. These organic acids have been studied using metabolic profiling as described in the following section.

### Metabolic profiling of mutants at different time-points throughout the light/dark cycle using HR-MAS NMR

**Fig 3:**
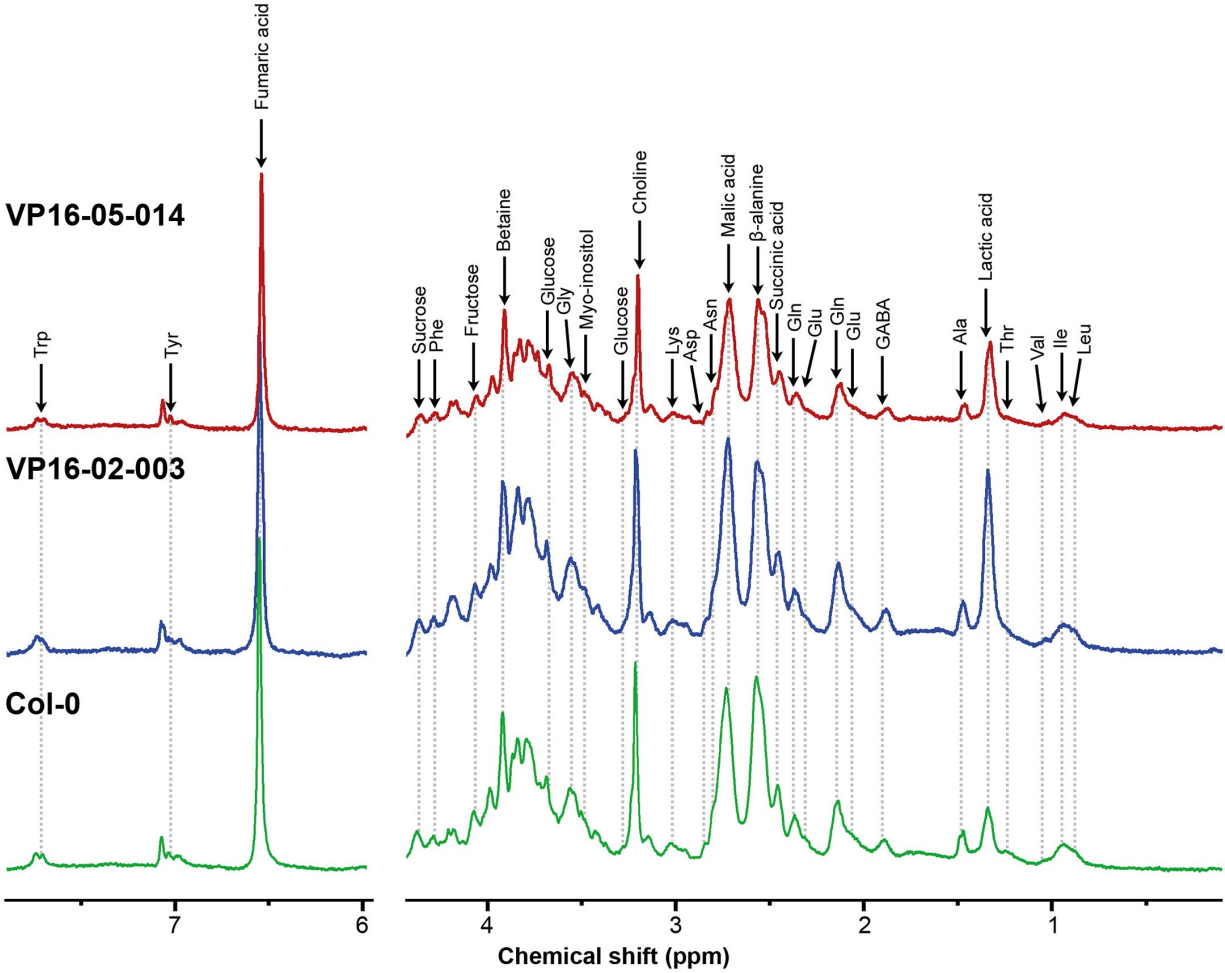
One-dimensional ^1^H CPMG NMR spectra for *Arabidopsis thaliana* Col-0 (bottom panels), VP16-02-003 (middle panels) and VP16-05-014 (top panels) obtained from intact rosette leaf harvested at t = 8 hours into the light/dark cycle. Main metabolites have been assigned in the spectra.

Metabolic profiles have been obtained from intact leaves of *Arabidopsis thaliana* at different time-points throughout the circadian cycle for the wild-type Col-0 and for both mutants with enhanced growth characteristics. Fig 3 shows representative one-dimensional ^1^H HR-MAS NMR spectra for Col-0, VP16-02-003 and VP16-05-014 at t = 8 hours.

**Fig 4:**
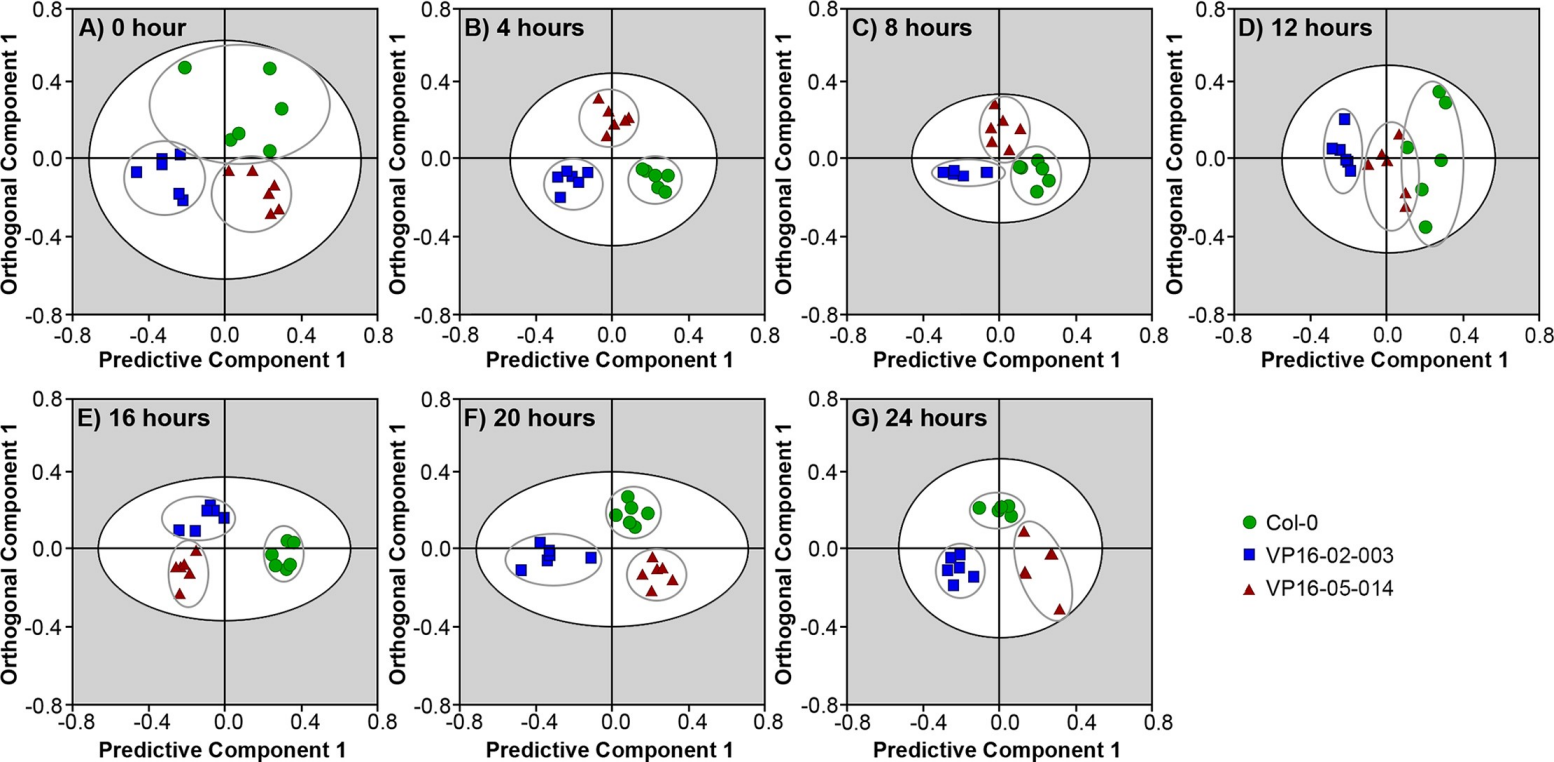
Orthogonal partial least square-discriminant analysis (OPLS-DA) score plots of the metabolite profiles derived from the intact leaves of *Arabidopsis thaliana* wild-type Col-0 (●), VP16-02-003 (■) and VP16-05-014 (▲) at the seven different time-points of harvesting throughout the light/dark cycle. There is a clear separation between the wild-type and the mutants at the different time-points.

The metabolic profiles for every time-point obtained by ^1^H HR-MAS NMR were investigated by multivariate analysis of the bucket-reduced ^1^H NMR data, as has been described in our earlier work [8]. In Fig 4, orthogonal partial least square discriminant analysis (OPLS-DA) was carried out to examine the variation in metabolic profile between two mutants and the wild-type Col-0 throughout the light/dark cycle. The variation of the OPLS-DA model was realized by a separation of the orthogonal and predictive component of the replicates collected from the Col-0, VP16-02-003 and VP16-05-014. The goodness-of-fit parameters of the OPLS-DA models can be found in S1 Table. The predictive component was correlated with the differences between the Col-0 and mutant classes at t = 4, 8, 12 and 16 hours, while the orthogonal component was correlated with differences between Col-0 and mutant classes at t = 0, 20 and 24 hours. Hence, in the OPLS-DA score plots, leads to clustering and separation of the replicates belonging to Col-0 and mutant classes at every time-point of the light/dark cycle (Fig 4).

**Fig 5:**
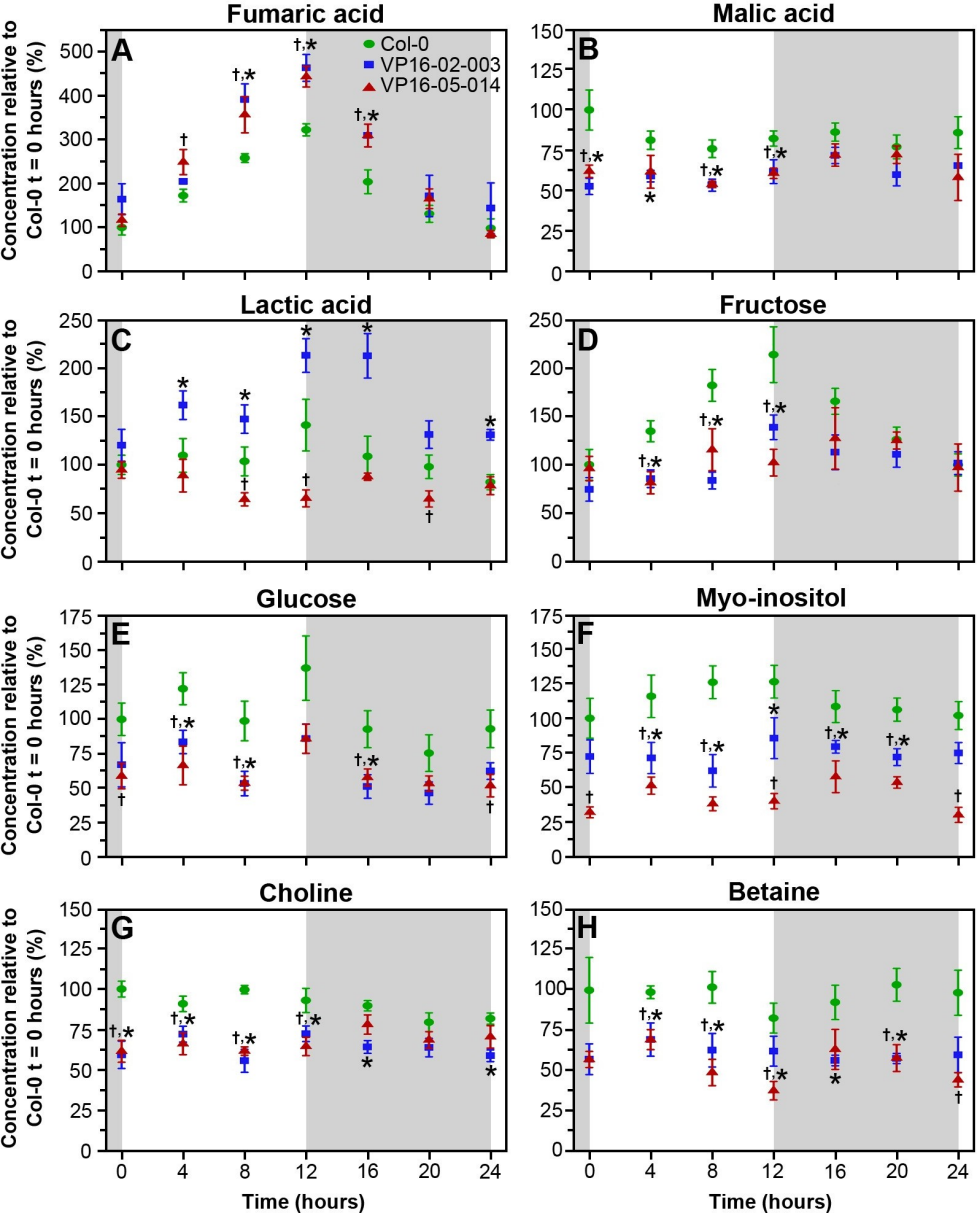
Metabolite concentrations during the circadian cycle of fumaric acid (A), malic acid (B), lactic acid (C), fructose (D), glucose (E), myo-inositol (F), choline (G) and betaine (H) in *Arabidopsis thaliana* Col-0 (●), VP16-02-003 (◼) and VP16-05-014 (▲). Means ± SEM of 6 biological replicates is shown. Concentrations are expressed relative to the concentration of Col-0 at t = 0 hours. * p < 0.05 Col-0 vs VP16-02-003, ^†^ p < 0.05 Col-0 vs VP16-05-014.

**Fig 6:**
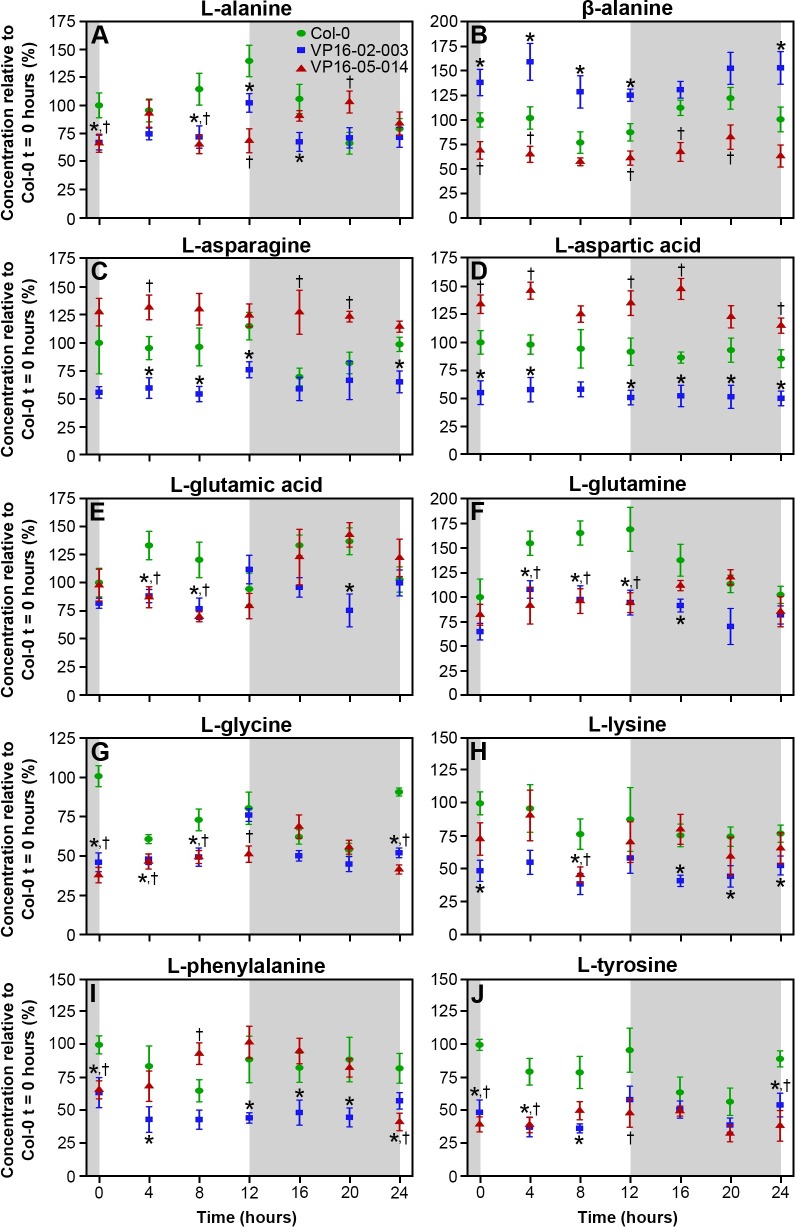
Concentration of the free amino acids L-alanine (A), 𝛃-alanine (B), L-asparagine (C), L-aspartic acid (D), L-glutamic acid (E), L-glutamine (F), L-glycine (G), L-lysine (H), L-phenylalanine (I) and L-tyrosine (J) throughout the circadian cycle in *Arabidopsis thaliana* Col-0 (●), VP16-02-003 (◼) and VP16-05-014 (▲). Means ± SEM of 6 biological replicates is shown. Concentrations are expressed relative to the concentration of Col-0 at t = 0 hours. * p < 0.05 Col-0 vs VP16-02-003, ^†^ p < 0.05 Col-0 vs VP16-05-014.

In our earlier metabolomics study of the VP16-02-003 and VP16-05-014, eighteen biomarkers were identified at t = 6 hours after the start of the light period [8]. In the present study, the rhythm of these biomarkers is followed during the circadian cycle. These metabolites are the organic acids fumaric acid, malic acid and lactic acid, the sugars fructose and glucose, the precursor of cell wall components choline, the secondary metabolites myo-inositol, a sugar alcohol, and the organic osmolyte betaine (Fig 5). Also, the rhythmic pattern of ten free amino acids was followed (Fig 6).

### Characterization of metabolic rhythms throughout the circadian cycle

The circadian rhythms of the concentration of the organic acids fumaric acid, malic acid and lactic acid are shown in Fig 5A–5C. In *Arabidopsis*, fumaric acid is generally considered the major form of fixed carbon and accumulates in the mitochondria and in the cytosol [24–26]. Fig 5A and 5B show that the concentrations of fumaric acid and malic acid increase during the light to a maximum at the end of the light period and decrease during the dark period in wild-type Col-0 and for both mutants [7,25]. Interestingly the concentration of fumaric acid was significantly higher in both mutants as compared to Col-0, especially at the end of the light period (t = 12 hours). The rhythm pattern of the malic acid concentration in VP16-02-003 and VP16-05-014 also remains the same as for the wild-type Col-0, however, the concentration of malic acid was lower in both mutants as compared to Col-0 at all time-points. In our earlier study, we found that the concentration of lactic acid is high at the end of the light period and dropped during the dark period in *Arabidopsis thaliana* Col-0 [7]. VP16-02-003 follows the same rhythm but at a higher concentration, while VP16-05-014 has a lower lactic acid concentration in comparison to Col-0 at all time-points (Fig 5C).

Sugars are known to play an important role in the circadian clock, mainly by regulation of diurnal genes [27]. In *Arabidopsis*, the photosynthetic carbon fixation during the light period also produces sugars that can be used during the dark period [20]. Fig 5D and 5E show the concentration of fructose and glucose during the light/dark cycle. The concentration of fructose as well as glucose increased slightly during the light period and declined during the dark period in Col-0 and in both mutants. The overall concentration of both sugars was lower in VP16-02-003 and VP16-05-014 during the whole light/dark cycle as compared to Col-0. The results show that both mutants VP16-02-003 and VP16-05-014 use organic acids as a storage form of carbon instead of sugars.

Myo-inositol in plants has diverse biological roles including phosphate storage, cell wall biogenesis and stress tolerance [28]. Fig 5F shows the circadian rhythm of myo-inositol for the wild-type Col-0, VP16-02-003 and VP16-05-014. The changes in the concentration of myo-inositol were found to be very minor over the entire light/dark cycle in Col-0 as well as in VP16-02-003 and the VP16-05-014 mutant. However, both mutants show a lower concentration of myo-inositol as compared to Col-0 throughout the whole cycle. The lower concentration of myo-inositol during the circadian cycle in wild-type *Arabidopsis thaliana* is in line with mass spectrometry data in a previous study [29].

Choline is an essential metabolite which is needed to synthesize membrane phospholipids in plants. Choline can be oxidized to the organic osmolyte betaine [30,31]. For Col-0, the levels of choline decreased during the light period and increased during the dark period (Fig 5G). The VP16-02-003 and VP16-05-014 mutants again follow the same rhythm, however at a lower choline concentration than for Col-0 at all time-points. Similar to choline, the concentration of betaine decreased during the light period and increased during the dark period (Fig 5H) in the wild-type *Arabidopsis* Col-0, the VP16-02-003 and the VP16-05-014 mutant. However, the level of betaine was lower in both mutants compared to Col-0 throughout the circadian cycle.

Fig 6 shows the circadian rhythm for various amino acids levels for the wild-type Col-0 and the VP16-02-003 and VP16-05-014 mutants. Overall, the rhythmic pattern of the free amino acids during the circadian cycle does not differ between the mutants and the wild-type Col-0. The levels of alanine, glutamic acid, glutamine, glycine, lysine, phenylalanine and tyrosine were significantly lower in both mutants in comparison to Col-0 during whole circadian cycle. The concentrations of aspartic acid, asparagine and β-alanine were higher in VP16-02-003 and lower in the VP16-05-014 mutant in comparison to Col-0.

**Fig 7:**
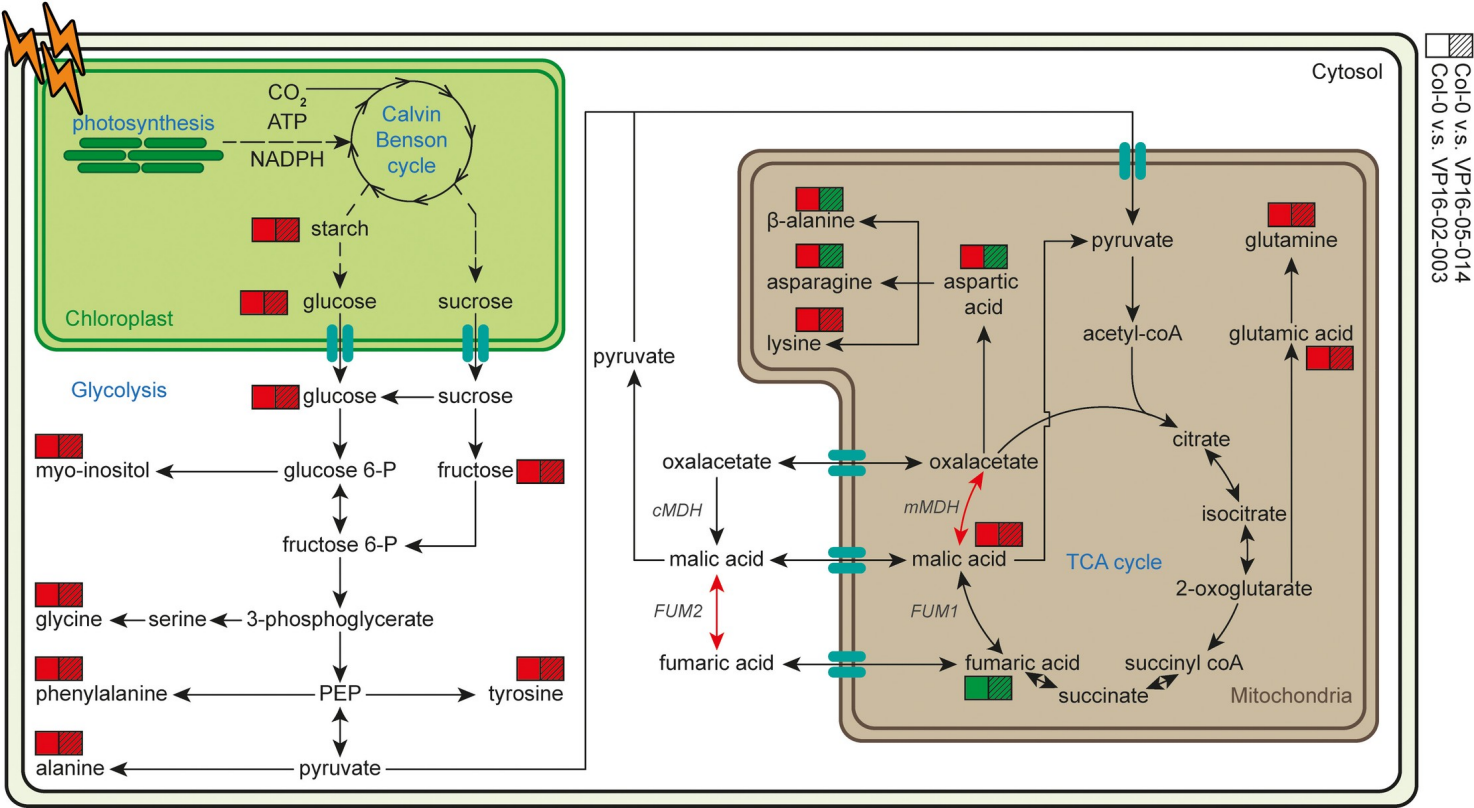
Pathway view of the central carbon metabolism of primary metabolites for the VP16-02-003 and VP16-05-014 mutant in comparison to Col-0. Colours indicate higher levels (green) or lower levels (red) of the primary metabolite. Dashed lines indicate multiple conversion steps.

Fig 7 summarizes the results of levels of the primary metabolites in the two mutants in comparison to Col-0 in the central carbon metabolism of *Arabidopsis thaliana*. What is remarkable is that only the concentration of fumaric acid increases for both mutants relative to Col-0, while the levels of the other primary metabolites are decreased for both mutants. The level of the amino acids aspartic acid, asparagine and β-alanine are higher in the VP16-05-014 while these amino acids are lower in the VP16-02-003 mutant in comparison to Col-0. Other detected free amino acids have lower concentration for both mutants in contrast to the wild-type. The lower concentrations of sugars, myo-inositol and free amino acids for these mutants compared to Col-0 were attributed to an impaired defence response in our previous study [8].

To better understand the high accumulation of fumaric acid, the TCA cycle has been studied in more detail by combining the metabolic data with the mRNA expression levels from the RNA-seq data [5]. The expression level of mitochondrial malate dehydrogenase 1 (mMDH1, AT1G53240) is reduced for both the VP16-02-003 and the VP16-05-014 mutant compared to Col-0. Malic acid can be converted to pyruvate in the mitochondria and in the cytosol. This conversion is facilitated by high concentrations of fumaric acid and may lead to increased fluxes through acetyl-coA back into the TCA cycle [23].

Fumaric acid and malic acid can be transported to the cytosol and can be interconverted in each other by cytosolic fumarase 2 (FUM2, AT5G50950) [32]. The mRNA expression level of FUM2 is reduced for both mutants in comparison to Col-0. The expression level of FUM2 for the VP16-02-003 mutant (logFC = -1.03) is even lower than for the VP16-05-014 mutant (logFC = -0.54). We have further analysed the enzyme activity of fumarase at t = 4 hours of the *Arabidopsis thaliana* Col-0, VP16-02-003 and VP16-05-014 mutant. As shown in Table 1, the activity of fumarase enzyme was significantly lower in both VP16-02-003 and the VP16-05-014 mutants as compared to Col-0. These results suggest that reduction in fumerase activity may be responsible for increased levels of fumaric acid observed in the VP16-02-003 and VP16-05-014. The natural *Arabidopsis* accession Rsch-4 has also reduced expression of FUM2 due to an insertion/deletion polymorphism in the promotor of the FUM2 gene and accumulates high levels of fumaric acid [32,33]. Next to the similarity in high levels of fumaric acid and reduced expression of FUM2, the rosette area of the *Arabidopsis* Rsch-4 was also larger in comparison to Col-0 [34].

**Table 1:** The enzyme activity of fumarase in the *Arabidopsis thaliana* wild-type Col-0 and the VP16-02-003 and VP16-05-014 mutant. Means ± SEM of 3 biological replicates is shown.

	Fumarase activity (nmol/gFW/min)
Col-0	99.7 ± 2.6
VP16-02-003	21.2 ± 8.9 *
VP16-05-014	77.9 ± 6.1 ^†^

* p < 0.05 Col-0 vs VP16-02-003

† p < 0.05 Col-0 vs VP16-05-014.

The relation between the high levels of fumaric acid and the occurrence of growth vigour is not yet clear. Earlier occurrences of growth vigour in *Arabidopsis* have been related to ploidy and hybridity effects subject to circadian regulation [35]. In addition, epigenetic regulation can lead to growth enhancement [36]. An example is the *Arabidopsis msh1* mutant where the nuclear-encoded MutS HOMOLOGUE 1 (MSH1) gene is downregulated. This triggers nuclear epigenetic reprogramming leading to enhanced growth vigour [36]. The zinc finger artificial transcription factors method is a very recent epigenetic reprogramming method to enhance growth [5,6].

For better understanding the relation between fumaric acid accumulation and growth vigour, and how primary metabolite concentrations are balanced in the interplay between photosynthetic source and utilization sinks leading to growth, examination of the enzyme activities of malate dehydrogenase and fumarase may lead to underpinning the underlying mechanisms of conversion of fumaric acid and malic acid [37]. Specific phenotypes can be examined by fluxomics where the metabolic fluxes are studied as the interplay of gene expression, protein concentration, kinetics, regulation and metabolite concentrations [38].

## Conclusion

In this study, we investigated the circadian rhythm of the eighteen earlier identified biomarker for the enhanced growth characteristics phenotype of the VP16-02-003 and VP16-05-014 mutant. HR-MAS NMR was used to obtain the metabolic profile throughout the light/dark cycle for *Arabidopsis thaliana* wild-type Col-0 and the VP16-02-003 and VP16-05-014 mutant. The metabolic rhythm of the eighteen biomarkers was not altered in both mutants as compared to Col-0, while the concentrations of metabolites differ significantly throughout the whole light/dark cycle. Since the clock functional periodicity is independent of the cellular complexity and growth-defence trade-off, the results contribute to converging evidence that it may not be necessary to—upstream—alter the circadian clock when the goal is to achieve enhanced growth characteristics, and that—downstream—phenotypic engineering of sinks and bottlenecks leading to growth may be more effective in a multifactorial context that can be altered by whole genome reprogramming.

## Supporting information

S1 Table The goodness-of-fit parameters R2X, R2Y and Q2 for the OPLS-DA models. (DOCX)

S1 Appendix NMR data files. (ZIP)
