# Supplementary material for: A robust circadian rhythm of metabolites in Arabidopsis thaliana mutants with enhanced growth characteristics
Source: PLoS One. 2019 Jun 25;14(6):e0218219. doi: 10.1371/journal.pone.0218219 (PMC6592530; doi:10.1371/journal.pone.0218219)
Supplement: S1 Table — (DOCX) [file pone.0218219.s001.docx]

## Supplementary information

**S1 table: The goodness-of-fit parameters R2X, R2Y and Q2 for the OPLS-DA models.**

|  | t = 0 h | t = 4 h | t = 8 h | t = 12 h | t = 16 h | t = 20 h | t = 24 h |
| --- | --- | --- | --- | --- | --- | --- | --- |
| R2X | 0.923 | 0.725 | 0.802 | 0.981 | 0.983 | 0.994 | 0.983 |
| R2Y | 0.991 | 0.909 | 0.869 | 0.999 | 0.891 | 0.998 | 0.999 |
| Q2 | 0.711 | 0.758 | 0.458 | 0.312 | 0.446 | 0.542 | 0.551 |
